# A Machine Learning Model Accurately Predicts Ulcerative Colitis Activity at One Year in Patients Treated with Anti-Tumour Necrosis Factor α Agents

**DOI:** 10.3390/medicina56110628

**Published:** 2020-11-20

**Authors:** Iolanda Valentina Popa, Alexandru Burlacu, Catalina Mihai, Cristina Cijevschi Prelipcean

**Affiliations:** 1 Department of Internal Medicine, University of Medicine and Pharmacy “Gr. T. Popa”, 700115 Iasi, Romania; iolivp@gmail.com (I.V.P.); alburlacu@yahoo.com (A.B.); 2 Head of Department of Interventional Cardiology—Cardiovascular Diseases Institute, 700503 Iasi, Romania; 3 Romanian Academy of Medical Sciences, 030167 Bucharest, Romania; 4 Institute of Gastroenterology and Hepatology, 700111 Iasi, Romania; cristinacijevschi@yahoo.com

**Keywords:** inflammatory bowel diseases, artificial intelligence, biological therapy, predictive model, disease activity

## Abstract

*Background and objectives:* The biological treatment is a promising therapeutic option for ulcerative colitis (UC) patients, being able to induce subclinical and long-term remission. However, the relatively high costs and the potential toxicity have led to intense debates over the most appropriate criteria for starting, stopping, and managing biologics in UC. Our aim was to build a machine learning (ML) model for predicting disease activity at one year in UC patients treated with anti-Tumour necrosis factor α agents as a useful tool to assist the clinician in the therapeutic decisions. *Materials and Methods:* Clinical and biological parameters and the endoscopic Mayo score were collected from 55 UC patients at the baseline and one year follow-up. A neural network model was built using the baseline endoscopic activity and four selected variables as inputs to predict whether a UC patient will have an active or inactive endoscopic disease at one year, under the same therapeutic regimen. *Results:* The classifier achieved an excellent performance predicting the disease activity at one year with an accuracy of 90% and area under curve (AUC) of 0.92 on the test set and an accuracy of 100% and an AUC of 1 on the validation set. *Conclusions:* Our proposed ML solution may prove to be a useful tool in assisting the clinicians’ decisions to increase the dose or switch to other biologic agents after the model’s validation on independent, external cohorts of patients.

## 1. Introduction

Ulcerative colitis (UC) is an inflammatory bowel disease (IBD) with recurrent and remissive evolution. Although the induction of a complete resolution of the disease is not currently possible, UC patients can benefit from the new biological therapies. Anti-Tumor Necrosis Factor α (anti-TNF) agents and other modern biological regimens ensured the success of the “treat to target” approach in UC [1] by their ability to achieve subclinical (endoscopic and histologic) remission [2], corticosteroid therapy discontinuation, reduction in hospitalization and surgery rates, long-term remission, and a good quality of life [3,4].

However, the high costs of biological agents and their potential side effects (mostly related to opportunistic infections and malignancies) [5] have led to intense debates over the most opportune timing for starting or discontinuing the therapy, increasing the dose, or switching to another biological regimen and deciding on the most appropriate management of the lack or loss of response [6,7,8].

Recent studies proved that anti-TNF antibodies are a practical approach for inducing clinical remission in UC [9]. Although promising results, evidence shows that long-term remission is only achieved in a certain proportion of the patients that initially responded to the treatment [10,11]. Besides, there is insufficient evidence to be sure of the ideal duration of the treatment [12]. The current “status quo” urges further research on optimizing the administration of anti-TNF antibodies. Additional studies for UC patients receiving biological therapy are needed to establish objective criteria for therapeutic decisions.

Modern artificial intelligence/machine learning (ML) solutions may fill this knowledge gap by in-depth investigation of the large clinical datasets available. To date, several innovative ML-based approaches [13] were proposed for various medical topics concerning IBD, such as endoscopic image analysis for the detection of inflammation [14], dysplasia [15], histologic disease activity [16], and disease subtype classification [17,18].

Various studies described ML methods for predicting IBD prognosis. Transcriptomic analyses on purified CD8 T cells and/or whole blood successfully predicted poor prognosis with earlier need for treatment escalation [19]. Other multi-omics ML approaches have been described for predicting IBD treatment outcomes with good performance [20,21]. However, multi-omics techniques are based on costly investigations that are not widely available, making them hardly applicable for routine use. Promising ML solutions for predicting disease outcome after treatment with Vedolizumab [22] or Azathioprine [23] based on standard clinical parameters have been proposed in IBD.

To date, no study based on routinely available clinical data has described ML models for predicting anti-TNF response in UC patients.

We aim to build a machine learning model for predicting disease activity and risk of relapse at one year follow-up in UC patients treated with anti-TNF agents using only standard clinical variables. After the model’s validation on independent, external, and sufficiently large cohorts of patients, the proposed ML solution may prove useful in assisting the clinicians’ decisions to increase the dose or change the biological agent.

## 2. Materials and Methods

### 2.1. Study Design and Participants

An observational retrospective single-centre cohort study was conducted on a sample of 55 UC patient records. All patients were admitted to the Institute of Gastroenterology and Hepatology, “Sf. Spiridon” Hospital Iași—Romania, between January 2012 and November 2018. Confirmed UC patients under maintenance therapy with an anti-TNF agent (Infliximab/Adalimumab) who underwent a colonoscopy for disease assessment at the initial evaluation and one year follow-up were considered. Only patients in clinical remission at the initial evaluation were included. Patients were excluded if they were in evidence with concurrent disorders (infections, autoimmune and inflammatory conditions, cirrhosis, neoplasia, and hemodialysis) capable of influencing medical parameters, if they presented clinical relapse at the initial evaluation or if changes were made to the therapeutic regimens between the two visits.

All patients provided written informed consent. The study has full ethical approval from the Research Ethics Commission of the “Gr. T. Popa” University of Medicine and Pharmacy (no. 15308/07.2019) and “St. Spiridon” Regional Hospital Ethics Committee (no. 54/10.2019). No sex-based or racial/ethnic-based differences were present.

### 2.2. Clinical Protocol

UC patients were hospitalized for treatment monitoring. According to the European consensus guidelines, UC diagnosis is established by clinical, biochemical, stool, endoscopic, cross-sectional imaging, and histological investigations [24]. Patients underwent a medical history interview, physical examination, routine laboratory tests, and colonoscopy to diagnose or assess already diagnosed UC, following the European standard protocols.

All included patients were in clinical remission and under maintenance therapy with an anti-TNF agent: Infliximab (5 mg/kg every eight weeks) or Adalimumab (40 mg every two weeks). No patient received other concomitant immunomodulatory treatment.

### 2.3. Data Collection

The following data were collected both at the initial evaluation and the one year follow-up.

Laboratory parameters documented were: red blood cells (RBC), white blood cells (WBC), platelets (PLT), hemoglobin (HGB), hematocrit (HCT), mean corpuscular hemoglobin concentration (MCHC), plateletcrit (PCT), platelet distribution width (PDW), mean platelet volume (MPV), platelet large cell ratio (PLCR), neutrophils (NEUT), lymphocytes, monocytes (MONO), C reactive protein (CRP), erythrocyte sedimentation rate/1 h (ESR), fibrinogen, serum iron (SI), ferritin, total proteins (TP), albumin, alpha one globulins (A1G), alpha two globulins (A2G), beta one globulins, beta two globulins, and gamma globulins.

Colonoscopy with biopsy was performed on the EVIS EXERA II endoscopy system (Olympus America). Specialist physicians carried out the procedures from Gastroenterology and Hepatology Institute, Iași, Romania. According to the colonoscopy findings, the Mayo subscore used to classify endoscopic disease activity [25,26] was documented. A patient was considered to have endoscopic remission if the Mayo score was 0 or 1. Similarly, active disease was considered for Mayo scores 2 or 3.

### 2.4. Preprocessing and Management of Missing Values

Documented continuous variables (biological parameters) were standardized in the range (0–1). Values of HGB, HCT, SI, and ferritin were processed to resolve the differences between sexes.

Missing values were assigned using multivariate imputation by chained equations (MICE) method implemented by the MICE package in R Studio Version 1.2.1335 © 2020–2019 RStudio, Inc. Build 1379 (f1ac3452). Missing variables were assigned by applying the Bayesian regression built-in method.

### 2.5. Standard Statistics for Feature Selection

ANOVA with Holm adjustments in R Studio was used to determine the continuous baseline variables for significant differences between the one year endoscopic remission and active groups. Statistical significance was considered for *p* < 0.05. If any two of the selected continuous variables had high intercorrelation with a Pearson coefficient ≥0.9, one of them was removed.

### 2.6. Neural Network Model—Construction and Evaluation

Initial data of 50 UC patient records were randomly divided into a training set of 40 records (80%) and a test set of 10 records (20%), such that variables distributions in each set were similar to those in the original dataset. The other five patient records from the same medical centre were added independently to be used as a validation set. Endoscopic activity classes (active/inactive) were not equally represented in the train and test set. However, the validation set had a balanced distribution of the disease activity classes.

One multilayered perceptron classifier was developed based upon the training set. The classifier was constructed using the caret: train function in R Studio. A 10-fold cross-validation was used to reduce overfitting. Synthetic minority over-sampling technique (SMOTE) was used with caret: train function to overcome imbalanced data.

The classifier was built to predict whether a UC patient will present endoscopic remission or active disease at the one year follow-up if the therapeutic strategy is left unchanged.

The developed neural network was evaluated on the test set and validation set according to the classification accuracy (ACC). The area under the receiver operating characteristic curve (AUC), sensitivity (SE), specificity (SP), positive and negative predictive values (PPV and NPV) were also determined.

## 3. Results

### 3.1. Patient Characteristics

Of all 55 patient records, 40 (72%) were males and 15 (28%) females. The age range of the participants was 22–62. The distribution of active/inactive endoscopic classes at the one year follow-up was imbalanced: 42 UC patients were reported with endoscopic remission and 13 with active disease.

### 3.2. Feature Selection

**Figure 1:**
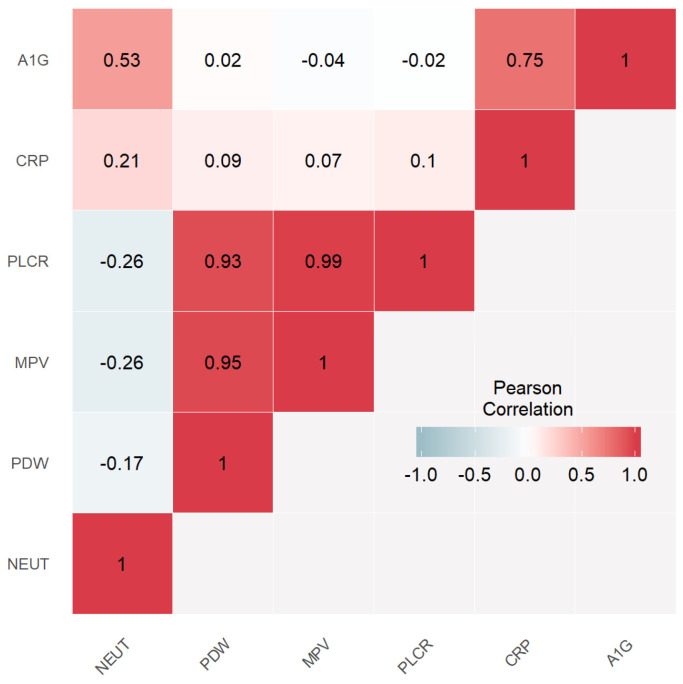
Correlation heatmap showing the Pearson coefficients between all parameters nominated by the feature selection method. NEUT (neutrophils), PDW (platelet distribution width), MPV (mean platelet volume), PLCR (platelet large cell ratio), CRP (C reactive protein), alpha one globulins (A1G).

Using ANOVA with Holm adjustment, the feature selection step initially included six continuous baseline variables with a significant difference between the active disease group and the remission group at one year: NEUT, PDW, MPV, PLCR, CRP, and A1G. Significance was established at *p* < 0.05. Next, highly intercorrelated features were identified and removed. Three strong correlations with a Pearson coefficient ≥0.9 were identified between PDW and MPV, PDW, and PLCR, MPV, and PLCR (Figure 1). Thus, the following parameters were removed from the analysis: MPV and PLCR.

As a result of the feature selection stage, four parameters (NEUT, PDW, CRP, and A1G) were included in further analysis.

Selected clinical characteristics and laboratory findings for all patient records and each activity class are summarized in Table 1.

### 3.3. Handled Missing Values

UC patient records had a total of 18 (8.2%) missing values, which were imputed using the MICE package as follows: PDW-4, CRP-2, A1G-12.

### 3.4. Results of the Neural Network Models Construction and Evaluation

Based on the results produced by the feature selection method, a neural network model was trained. The initial dataset of 50 UC patient records was randomly divided into a training set (40 records) and a test set (10 records) to build the classifier. Five patient records were added independently to constitute the validation set. Unlike in the training and test set, endoscopic activity classes were balanced in the validation set.

**Figure 2:**
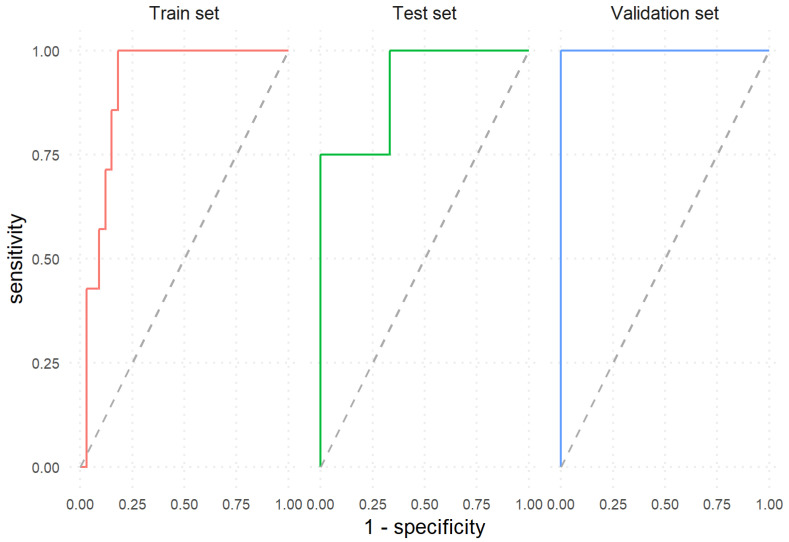
Classifier’s performance to predict endoscopic remission vs. relapse at one year.

The neural network model was developed using the baseline endoscopic activity and all four selected variables as inputs to predict whether a UC patient will have an active or inactive endoscopic disease at one year, under the same therapeutic regimen. Model performance metrics are shown in Table 2. ROC curves proving model performance on the train, test, and validation sets are shown in Figure 2.

## 4. Discussion

Our study is the first neural network developed for UC patients treated with anti-TNF agents for predicting endoscopic disease activity and risk of relapse at one year using standard baseline parameters. We demonstrated that it is possible to accurately predict anti-TNF response at one year in UC patients using machine learning methods. Present-day guidelines [25,26,27] indicate that the best future activity prediction is based on current endoscopic activity. Our solution brings a new perspective by incorporating additional parameters besides baseline endoscopy to predict future disease outcomes.

Measuring endoscopic disease activity is the “gold” standard for disease monitoring in UC according to current guidelines [25,26,27]. Indeed, strong evidence shows that targeting endoscopic healing is superior to tailing only clinical remission (concerning relapse rates, hospitalization rates, and the need for surgery) [28]. The advent of biological therapy in UC paved the way for achieving more profound, subclinical remission degrees (mucosal and histological healing). However, the inadequate response to biologics delays the disease’s resolution, exposes patients to unnecessary toxic drug effects, and wastes medical resources. Therefore, the growing interest in monitoring the therapy aims to identify appropriate end-points for successful treatment and timely discontinue or switch the therapy in those likely to relapse or unlikely to respond [29]. The proper therapeutic decision is essential, even more so as inopportune discontinuation may trigger the development of anti-drug antibodies that can lead to future response loss [30].

We aimed to develop a machine learning tool capable of predicting the endoscopic disease activity at one year. Comparing the baseline activity with the predicted one and acknowledging the risk of relapse, the clinician may decide whether increasing the dose, switching to other biologic agents, or discontinuing the therapy is the most appropriate decision. Our model achieved an ACC of 85% with 82% SE, 100% SP and an AUC of 0.91 on the trainset. On the test set, the classifier obtained an excellent performance with an ACC of 90%, SE of 100%, SP of 75%, and AUC of 0.92. On the validation set, the model predictions achieved the maximum performance with a 100% ACC, SE, and SP and an AUC of 1.

A few other studies aimed to predict endoscopic remission at one year. One paper used faecal calprotectin (FC) assay measured after the induction of anti-TNF therapy (infliximab) to predict the mucosal healing after one year of treatment [31]. A cut-off of ≤121 μg/g used for the post-induction faecal calprotectin achieved 70% SE and 70% SP for predicting mucosal healing at one year in 50 patients with UC colonic or ileal-colonic Crohn’s disease. Our model obtained higher performance metrics on all tested sets.

A particular aspect worth mentioning is that, in our study, baseline CRP levels differed significantly in patients developing an active disease versus patients in endoscopic remission at one year. Our result is consistent with the findings of other studies. CRP was shown to be a clinically relevant biomarker of response to infliximab [32] and an independent predictor of colectomy-free survival in patients treated with infliximab [33].

### Limitations and Future Perspectives

Firstly, our dataset’s minimal size and the fact that the independent validation set is from the same centre entails rigorous external validation with data from other centres. Secondly, the imbalanced distribution of endoscopic activity classes at one year predisposes to calculation biases, although the SMOTE function in R was used to reduce these biases significantly. Thirdly, the retrospective nature of our study may introduce further bias.

In the future, these drawbacks could be overcome by employing prospective studies on broader, more diverse, and comprehensive datasets in a centre with greater accessibility that would permit organizing a cohort with a balanced distribution of endoscopic activity classes both at baseline and at one year. The next trials would improve models’ performance using different ML algorithms as our patient’s database extends.

## 5. Conclusions

Our proposed ML solution proved to accurately predict disease activity at one year in UC patients treated with anti-TNF agents using routinely available clinical parameters. Acknowledging the risk of relapse could lead to increasing the dose or switching to other biological agents. After rigorous validation on large, external datasets, our ML approach could significantly impact clinical practice by helping the physician decide on the most appropriate therapeutic option concerning the management of anti-TNF biologics.

## Figures and Tables

**Table 1:** Baseline parameters for all patient records and each endoscopic activity class at one year.

Baseline Parameters	All		Endoscopic Activity at One Year	
			Inactive	Active
Number of records	55		42	13
Gender (male:female)	40:15		32:10	8:5
Age (years)	44.3 ± 10.5		43.7 ± 11.4	46 ± 6.4
Baseline endoscopic activity	Inactive	39	35	4
	Active	16	7	9
NEUT * 10^3^/µL	4.59 ± 2		3.32 ± 1.13	5.7 ± 2.37
PDW fL	12.9 ± 1.9		13.2 ± 2.1	11.8 ± 1
CRP mg/dL	0.35 ± 0.4		0.3 ± 0.32	0.55 ± 0.4
A1G %	2.1 ± 0.33		2 ± 0.32	2.31 ± 0.27

* signifies “multiplied by”.

**Table 2:** The classifier’s performance metrics.

	Train Set		Test Set		Validation Set	
	Predictions		Predictions		Predictions	
Actual	Remission	Activity	Remission	Activity	Remission	Activity
Remission	27	0	6	1	3	0
Activity	6	7	0	3	0	2
ACC	85%		90%		100%	
95% CI	(0.70, 0.94)		(0.56, 0.99)		(0.48, 1.00)	
*p* value	<0.001		<0.001		<0.001	
SE	82%		100%		100%	
SP	100%		75%		100%	
PPV	100%		86%		100%	
NPV	54%		100%		100%	
AUC	0.91		0.92		1.00	

ACC (Accuracy); CI (Confidence Intervals); AUC (Area under the receiver operating characteristic curve); SE (Sensitivity); SP (Specificity), PPV (Positive predictive value); NPV (Negative predictive value).
